# Melatonin Reduces Aggravation of Renal Ischemia–Reperfusion Injury in Obese Rats by Maintaining Mitochondrial Homeostasis and Integrity through AMPK/PGC-1α/SIRT3/SOD2 Activation

**DOI:** 10.3390/cimb45100520

**Published:** 2023-10-11

**Authors:** Anongporn Kobroob, Aphisek Kongkaew, Orawan Wongmekiat

**Affiliations:** 1 Division of Physiology, School of Medical Sciences, University of Phayao, Phayao 56000, Thailand; anongporn.ko@up.ac.th; 2 Research Administration Section, Faculty of Medicine, Chiang Mai University, Chiang Mai 50200, Thailand; aphisek.k@cmu.ac.th; 3 Integrative Renal Research Unit, Department of Physiology, Faculty of Medicine, Chiang Mai University, Chiang Mai 50200, Thailand

**Keywords:** melatonin, mitochondria, ischemia–reperfusion, acute kidney injury, oxidative stress

## Abstract

This study examined the potential benefits of melatonin against renal ischemia and reperfusion (IR) injury in obesity and explored the underlying mechanisms. Obesity was induced in Wistar rats by feeding a high-fat diet for 16 weeks. Three obese groups that underwent renal IR induction (30-min renal ischemia followed by 24-h reperfusion) were randomly assigned to receive melatonin at ischemic onset, reperfusion onset, or pretreatment for 4 weeks before IR induction. Groups of vehicle-treated obese and normal-diet-fed rats that underwent sham or IR induction were also included in the study. The results showed that renal functional and structural impairments after IR incidence were aggravated in obese rats compared to normal-diet-fed rats. The obese-IR rats also exhibited oxidative stress, mitochondrial dysfunction, apoptosis, and mitochondrial dynamics and mitophagy imbalances, which were all considerably improved upon melatonin treatment, irrespective of the treatment time. This study suggests the prophylactic and therapeutic efficacy of melatonin in IR-induced acute kidney injury (AKI) in obese individuals, which may improve the prognosis of AKI in these populations. The benefits of melatonin are likely mediated by the modification of various signaling molecules within the mitochondria that maintain mitochondrial redox balance and lead to the protection of mitochondrial homeostasis and integrity.

## 1. Introduction

Acute kidney injury (AKI) is characterized by a sudden deterioration in renal function [1]. AKI is a globally prevalent health problem with rising incidence, multiple comorbidities, an increased long-term risk of mortality, and increased medical costs. Ischemia and reperfusion (IR) is well recognized as a major cause of AKI [2]. Due to their high metabolic rate, renal cells are susceptible to the harmful consequences of ischemia, including ATP depletion and hypoxia-related mitochondrial damage. Renal IR injury leads to cellular dysfunction, apoptosis, necrosis, and eventually organ dysfunction. In addition, injured kidneys remain at high risk of developing chronic kidney disease (CKD) after renal IR incidence, which may eventually progress to end-stage renal disease (ESRD) in the long run. Renal IR injury is therefore a complex pathophysiological process with myriad etiologies and is commonly encountered in various clinical settings, such as shock, congestive heart failure, transplantation, and vascular surgery. Oxidative stress, together with mitochondrial injury during the ischemia and reperfusion period, has been acknowledged as playing a crucial role in the pathogenesis of AKI [3]. Despite significant advances in preventive and therapeutic approaches, renal IR-induced AKI is still associated with unacceptably high mortality and effective strategies have yet to be further explored. Thus, treating acute renal IR injury remains a formidable challenge for clinicians in daily practice.

Obesity is a medical condition in which excess body fat is accumulated to the extent that it may have a negative effect on health [4]. The World Health Organization (WHO) has reported that more than 1.9 billion adults are overweight; of these, over 650 million adults and 100 million children are obese [4]. The prevalence of obesity continues to increase worldwide, and it is expected to reach 50% by the year 2030 [5]. Obesity is the major risk factor for the development of several non-communicable diseases, such as cardiovascular diseases and diabetes [5]. Importantly, obesity is now being increasingly recognized as a major and independent risk factor for the development of kidney disease. In the general population, obesity is the second most highly predictive factor in predicting ESRD, even independently of diabetes and hypertension [5].

In an obese state, the excessive accumulation of fat in the kidney results in lipotoxicity, which leads to various structural, hemodynamic, and metabolic alterations in the kidney. Most of these are likely compensatory responses to the increase in systemic metabolic demands associated with obesity, and kidney injury becomes clinically apparent because of compensatory failure [6,7]. The histological changes in the kidneys in obesity-induced renal injury include glomerular or tubular hypertrophy, focal segmental glomerulosclerosis, or bulbous sclerosis [6]. The increase in intraglomerular pressure owing to compensatory hyperfiltration can damage the kidneys and raise the risk of developing CKD in the long term. Furthermore, renal hemodynamic changes, insulin resistance, and lipid metabolism disorders are all involved in the development and progression of obesity-induced kidney injury [6]. Notably, recent studies have shown that obesity potentiates the frequency and severity of AKI [8,9].

Recent evidence suggests that oxidative stress may be the mechanistic link between obesity and its related complications. Studies have shown that high-fat-diet-induced obesity stimulates intracellular pathways, leading to increased reactive oxygen species (ROS) production through multiple biochemical mechanisms, such as superoxide generation from NADPH oxidases (Nox), glyceraldehyde autoxidation, protein kinase C (PKC) activation, and the polyol and hexosamine pathways [10]. ROS also trigger the release of inflammatory cytokines, which in turn enhances ROS production and establishes a vicious cycle. Growing evidence has demonstrated that tissue lipotoxicity also induces mitochondrial dysfunction and promotes ROS production in obesity through oxidative phosphorylation [10]. Thus, oxidative stress, inflammation, and mitochondrial dysfunction appear to be closely interlinked in obesity, although it is difficult to establish the temporal sequence of their relationship.

Current information reveals that more than 13.3 million people worldwide experience acute kidney injury each year, and the annual mortality rate of AKI exceeds 50% and is greater than those of breast and prostate cancer, heart failure, and diabetes together [11]. According to the increasing global epidemic of obesity, it is most likely that the incidence, severity, and mortality of AKI will be aggravated and novel strategies for the prevention and treatment of AKI in obese conditions is needed. In the context of the close association of AKI and obesity with oxidative stress and mitochondrial damage [10], an antioxidant that also possesses mitochondrial protection could be of great benefit.

Melatonin (N-acetyl-5-methoxytryptamine), an indoleamine found in all organisms, is an endogenous hormone that is released by the pineal gland and plays a key role in regulating many physiological functions, such as the circadian rhythm, reproductive cycle, and so on. Melatonin modulates cellular function through melatonin receptor-1 (MT1) and melatonin receptor-2 (MT2), which are widely distributed in the body, including the kidney, where both receptors are predominately expressed in the kidney membrane area and basolateral membranes [12]. Melatonin has several biological and pharmacological properties, particularly strong antioxidant activity. It has been demonstrated that not only melatonin itself but its derivatives are also highly efficient radical scavengers [13]. Melatonin has also been reported to chelate transition metals, thus reducing the formation of highly toxic hydroxyl radicals, and results in reduced oxidative stress [14]. In addition to increases in other antioxidant enzymes’ activity, melatonin boosts the mRNA expression and activity of several enzymes necessary for glutathione synthesis and recycling, thus increasing the availability of glutathione. All these properties of melatonin make it a more powerful antioxidant than other well-known antioxidants, such as vitamin E and vitamin C [15]. Studies have shown that melatonin has potential in the protection against several diseases that are related to oxidative stress [16,17]. Most importantly, mitochondria have been identified as a target for melatonin’s action [18]. Melatonin increases the efficiency of mitochondrial oxidative phosphorylation and reduces electron leakage, thereby lowering free radical generation [15]. Interestingly, recent studies have shown that treatment with melatonin reduced mitochondrial dysfunction caused by cardiac ischemia–reperfusion injury in type 1 diabetic rats [19] as well as prediabetic obese rats [20].

Based on the above information, it is possible that melatonin has the potential to reduce AKI caused by ischemia and reperfusion in obese individuals via its ability to sustain redox equilibrium and maintain mitochondrial function and integrity. The present study aimed at testing this hypothesis using a rat model of obesity induced by high-fat feeding. Signaling pathways involving mitochondrial homeostasis were also examined. The study outcomes provided basic insights into the mitochondrial protective role of melatonin during an acute renal ischemia–reperfusion episode, which may lead to new approaches for the future clinical management of AKI in obesity.

## 2. Materials and Methods

### 2.1. Animal Preparations

Forty-two male Wistar rats at the age of 6 weeks (130–150 g) were obtained from Nomura Siam International, Bangkok, Thailand, and housed under standard temperature and humidity conditions with a 12 h light–dark cycle. Standard commercial rat chow and water were given ad libitum. The animals were allowed one week to acclimatize before starting the experiment. All procedures were conducted in conformity with the guidance for the use of animals of the National Research Council of Thailand and approved by the Institutional Animal Care and Use Committee at the Faculty of Medicine, Chiang Mai University (project number 03/2562).

### 2.2. Induction of Renal Ischemia and Reperfusion

The surgery to induce renal ischemia and reperfusion (IR) was carried out as previously described [3] under zoletil and xylazine anesthesia (37.5 and 2.25 mg/kg, i.p., respectively). Briefly, both kidneys were approached through a midline incision and ischemia was induced by clamping both renal pedicles with non-traumatic vessel clips. The success of ischemic induction was confirmed by the occurrence of immediate blanching of the kidneys followed by a dusky color before temporarily closing the abdomen. The abdomen was reopened after 30 min of ischemia to allow for 24 h of reperfusion, the clamps were removed, the reperfusion was verified by full color restoration, and the abdominal cavity was sutured with silk 4-0. The animals were allowed to fully recover from the anesthesia before returning to their cage. For sham operation, rats underwent an identical anesthetic procedure and the exposure of both renal pedicles for a comparable duration without any occlusion. All surgical procedures were performed under the aseptic technique.

### 2.3. Experimental Protocols

**Figure 1:**
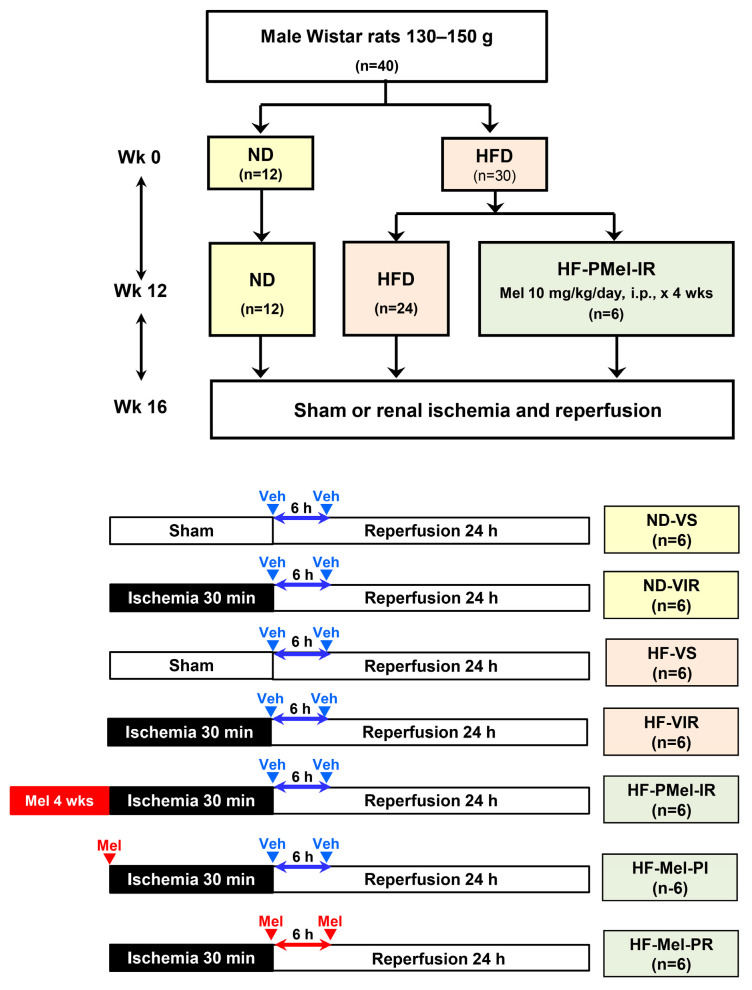
A schematic of the experimental design. ND-VS: normal-diet-fed rats and a vehicle-treated sham group; ND-VIR: normal-diet-fed rats and a vehicle-treated IR group; HF-VS: high-fat-fed rats and a vehicle-treated sham group; HF-VIR: high-fat-fed rats and a vehicle-treated IR group; HF-PMel-IR: high-fat-fed rats and melatonin pretreatment 4 weeks before IR induction; HF-Mel-PI: high-fat-diet-fed rats and melatonin treatment 5 min before ischemia; HF-Mel-PR: high-fat-diet-fed rats and melatonin treatment 5 min before and 6 h after reperfusion.

Figure 1 shows a schematic of the experimental design. Following acclimatization, thirty rats were fed a high-fat diet (HFD) for the whole 16-week experiment to induce obesity. Five groups of HFD-fed rats (n = 6 each) were studied. The HF-fed-vehicle-sham (HF-VS) and HF-fed-vehicle-IR (HF-VIR) groups received the vehicle and underwent sham or renal IR induction, respectively. The HF-Mel-PI group received melatonin (10 mg/kg, i.p.) 5 min before ischemia induction. The HF-Mel-PR groups were injected with the same dose of melatonin at the onset of reperfusion and again 6 h post-reperfusion. Melatonin was dissolved in dimethyl sulfoxide (DMSO) and diluted in normal saline solution to a final concentration of 0.5% DMSO (vehicle). The dose and regimen for melatonin treatment were adapted from studies showing the benefit of melatonin in ischemic stroke [21] and the suggested time required to boost the reduction in renal IR injury [22]. The last HF-fed group, HF-PMel-IR, was given melatonin (10 mg/kg/day, i.p.) pretreatment that started at week 12 and continued to week 16 before being subjected to renal IR induction. This group was used to study the prophylactic effect of melatonin supplementation on kidney injury after renal IR incidence. Two more groups (n = 6 each) fed a normal diet (ND) were also included, the ND-VS and ND-VIR groups, which were subjected to sham operation and IR induction, respectively. These two ND groups were used to evaluate the success of obesity induction by HFD feeding and examine the impact of HFD on kidney injury after ischemia and reperfusion. After full recovery from the surgery, each rat was placed in a metabolic cage throughout the reperfusion period to collect urine for the determination of renal function. At the end of urine collection, a blood sample was taken via the abdominal aorta under thiopental (60 mg/kg, i.p.) anesthesia. Both kidneys were quickly removed. One portion of the kidney was immediately taken to test mitochondrial function, other pieces were fixed in the proper fixative solutions for histopathological exams, and the remaining parts were snap-frozen in liquid nitrogen and kept at −80 °C for further analyses.

### 2.4. Determination of Serum Cholesterol and Triglycerides

Serum levels of total cholesterol and triglycerides were determined using an AU480 Chemistry Analyzer (Beckman Coulter Inc., Brea, CA, USA).

### 2.5. Assessment of Renal Function

Serum samples were analyzed for urea nitrogen (BUN) and creatinine and urine samples were analyzed for creatinine using an AU480 Chemistry Analyzer (Beckman Coulter Inc., Brea, CA, USA). Creatinine clearance was calculated using the standard clearance formula.

### 2.6. Light Microscopic Studies

The kidney tissues fixed in 10% neutral formaldehyde buffered were dehydrated through a graded alcohol series, cleared in xylene, and embedded in paraffin wax. Serial sections of 4 μm were deparaffinized, hydrated, and stained with hematoxylin and eosin (H&E) for light microscopic examination.

### 2.7. Transmission Electron Microscopic Studies

The electron microscopic technique was performed as described previously [3]. Briefly, kidney tissues were fixed overnight with 2.5% glutaraldehyde in 0.1 M phosphate buffer (pH 7.4, 4 °C), post-fixed in 2% phosphate-buffered osmium tetroxide, dehydrated with graded ethanol, rinsed in propylene oxide, and embedded in Epon resin using the EMbed-812 embedding kit (Electron Microscopic Sciences, Hatfield, PA, USA). Sections of 60–80 nm thickness were mounted on copper grids, stained with uranyl acetate followed by lead citrate, and examined using a JEM-2200 FS transmission electron microscope (JEOL, Tokyo, Japan).

### 2.8. Assessment of Renal Oxidative Stress

The kidney tissues were homogenized in an appropriate buffer using a Potter Elvehjem glass–Teflon homogenizer (Wheaton Science, Millville, NJ, USA). The tissue homogenate was centrifuged at 10,000× *g* for 15 min at 4 °C, and the supernatants were collected for the determination of nitric oxide (NO), malondialdehyde (MDA), superoxide dismutase (SOD), and glutathione (GSH) using commercial kits obtained from Bioassay Systems (Hayward, CA, USA), according to the manufacturer’s instructions.

### 2.9. Mitochondrial Studies

#### 2.9.1. Preparation of Mitochondrial Fractions and Mitochondrial Proteins

Kidney tissues were homogenized in cold lysis buffer (230 mM mannitol, 70 mM su-crose, 1 mM EDTA, and 10 mM Tris–HCl, pH 7.4) and mitochondria were isolated by differential centrifugation as previously described [23]. The final mitochondrial pellet was suspended in an ice-cold respiration buffer (250 mM sucrose, 5 mM KH_2_PO_4_, 10 mM Tris–HCl, 2 mg/mL BSA, pH 7.2), and a bicinchoninic acid (BCA) assay was used to quantify the mitochondrial protein content.

#### 2.9.2. Determination of Mitochondrial ROS

Mitochondrial ROS were assayed using a cell-permeable fluorogenic probe, 2′,7′-dichlorofluorescein diacetate (DCFDA), as previously described [23]. Briefly, mitochondria were stained with 2 mM DCFDA for 60 min at 25 °C. The fluorescence emission from DCF was determined by a fluorescence microplate reader with excitation/emission spectra set at 485/530 nm, respectively. The levels of ROS were expressed in arbitrary units of DCF fluorescence intensity.

#### 2.9.3. Determination of Mitochondrial Membrane Potential

A lipophilic cationic fluorescence dye, 5,5′,6,6′-tetrachloro-1,1′,3,3′-tetraethylbenzimi-dazocarbocyanine iodide (JC-1), was used to assess the mitochondrial membrane potential (∆Ψm) change according to the method described previously [23]. Briefly, mitochondria were stained with 310 nM JC-1 and incubated at 37 °C for 30 min. A fluorescence microplate reader was used to detect the J-aggregate form of JC-1 using excitation of 485 nm and emission at 590 nm, as well as the monomeric form of JC-1 at excitation and emission wavelengths of 485 and 530 nm, respectively. Changes in ∆Ψm reflected by different forms of JC-1 were quantified, and mitochondrial depolarization was represented by a decrease in the red/green fluorescence intensity ratio.

#### 2.9.4. Determination of Mitochondrial Swelling

Mitochondria swelling was determined by measuring the change in mitochondrial absorbance at 540 nm using a microplate reader (Synergy^TM^ H4, BIOTEKÒ Instruments, Inc., Winooski, VT, USA) [17]. The swelling of mitochondria was indicated by a decrease in absorbance over 15 min.

### 2.10. Western Blot Analysis

Renal cortical tissues were homogenized in lysis buffer containing 20 mM Tris (pH 6.8), 1 mM sodium orthovanadate, 5 mM sodium fluoride, and protease inhibitor. All protein concentrations were examined using a Bio-Rad protein assay kit (Bio-Rad Laboratories, Hercules, CA, USA). Aliquots of protein lysates were separated on 10% SDS-PAGE gels, blotted onto nitrocellulose membranes (Millipore, Burlington, MA, USA), and blocked with 5% skim milk or bovine serum albumin in 0.1% Tris-buffered saline and Tween (TBST). The membranes were incubated at 4 °C overnight with primary antibodies against AMP-activated protein kinase (AMPK), sirtuin 3 (SIRT3), superoxide dismutase 2 (SOD2), B-cell lymphoma 2 (Bcl-2), pro-caspase3, cleaved-caspase3, dynamin-like protein 1 (Drp1), phospho-dynamin-like protein 1 at Ser616 (p-Drp1Ser616), mitofusin 2 (Mfn2), PTEN-induced putative kinase 1 (PINK1), voltage-dependent anion channel (VDAC), PARKIN, β-actin (Cell Signaling Technology, Danvers, MA, USA), peroxisome proliferator-activated receptor gamma coactivator 1 alpha (PGC-1α), phospho-AMPK at Thr172 (p-AMPKThr172) (Millipore Corporation, Burlington, MA, USA), acetylated SOD2 (Ac-SOD2), and Bcl-2-associated X protein (Bax) (Abcam, Cambridge, MA, USA), followed by the corresponding secondary antibodies. Protein bands were detected using the ChemiDoc Touch Imaging System (Bio-Rad Laboratories, Hercules, CA, USA) and densitometric analysis was performed by the ImageJ program (National Institute of Health, Rockville, MD, USA).

### 2.11. Statistical Analysis

Data are expressed as means ± SEM. Statistical analysis was performed using one-way analysis of variance (ANOVA) followed by Fisher’s post-hoc test or the nonparametric Kruskal–Wallis test (as appropriate). *p* < 0.05 was considered statistical significance. All analyses were performed using the SPSS software version 25 (IBM Corporation, Armonk, NY, USA).

## 3. Results

### 3.1. Long-Term HFD Feeding Successfully Induced Obesity

The initial body weights of all rats were very similar (Table 1). After 16 weeks of dietary management, all high-fat diet (HFD)-fed rats exhibited a noticeable increase in body weight gain, which was almost three times higher than their initial values (*p* < 0.05). The increase rate was significantly higher than that detected in the normal diet (ND)-fed rats. Moreover, the HFD rats exhibited significant increases in serum triglycerides and total cholesterol levels compared to the ND rats. All these data verified the successful induction of the obesity model in our study.

### 3.2. IR-Induced Renal Injury Is Aggravated in HFD-Fed Rats and Melatonin Treatment Attenuates Renal Functional and Histopathological Alterations Caused by IR

**Figure 2:**
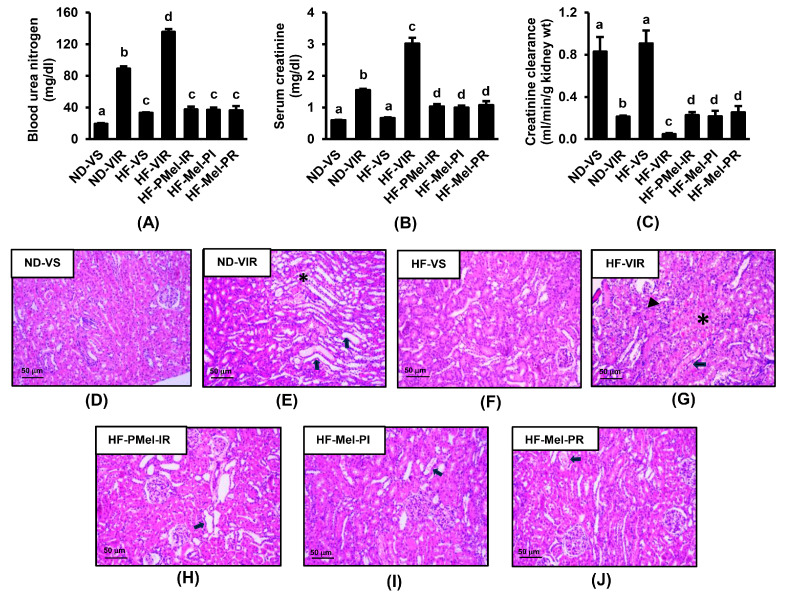
Effects of melatonin treatment on renal function and histopathological alterations following ischemia–reperfusion (IR). (**A**) Blood urea nitrogen; (**B**) serum creatinine; (**C**) creatinine clearance; and (**D–J**) H&E staining of kidney sections (40×) showing tubular dilatation and obstruction (arrow), apoptotic and necrosis (asterisk), and glomerular atrophy (arrowhead). Values are means ± SEM (n = 6 each). ND-VS: normal-diet-fed rats and a vehicle-treated sham group; ND-VIR: normal-diet-fed rats and a vehicle-treated IR group; HF-VS: high-fat-fed rats and a vehicle-treated sham group; HF-VIR: high-fat-fed rats and a vehicle-treated IR group; HF-PMel-IR: high-fat-diet-fed rats and melatonin pretreatment 4 weeks before IR induction; HF-Mel-PI: high-fat-diet-fed rats and melatonin treatment 5 min before ischemia; HF-Mel-PR: high-fat-diet-fed rats and melatonin treatment 5 min before reperfusion. Different lowercase letters denote statistical differences at *p* < 0.05 between groups.

To assess the impact of obesity on IR-induced renal injury, both ND-fed and HFD-fed rats were subjected to sham operation or renal IR induction. The effect of melatonin on IR-induced renal injury in obesity was also assessed in three HFD groups, each receiving melatonin at different times during IR induction. The results demonstrated that all rats that underwent renal IR induction exhibited renal dysfunction, as evidenced by significant increases in blood urea nitrogen and serum creatinine together with a decrease in creatinine clearance compared to their respective sham controls (Figure 2). The severity of IR-induced renal dysfunction was much greater in the group of HF-VIR rats than in the ND-VIR rats. Histopathological analysis of the kidneys from both ND-VIR and HF-VIR rats revealed proximal tubular damage characterized by brush border loss, tubular dilatation, cast formation, and tubular obstruction. However, numerous apoptotic cells and severe tubular necrosis were detected only in the HF-VIR group. The findings provided further support for the aggravation of renal IR injury by long-term HFD feeding. Most importantly, treatment with melatonin in all obese-IR rats, regardless of the time administered, significantly attenuated the renal functional and histopathological alterations induced by renal IR.

### 3.3. Melatonin Ameliorates Renal IR Injury in Obese Rats via the Maintenanace of Oxidative Balance within the Kidneys

**Figure 3:**
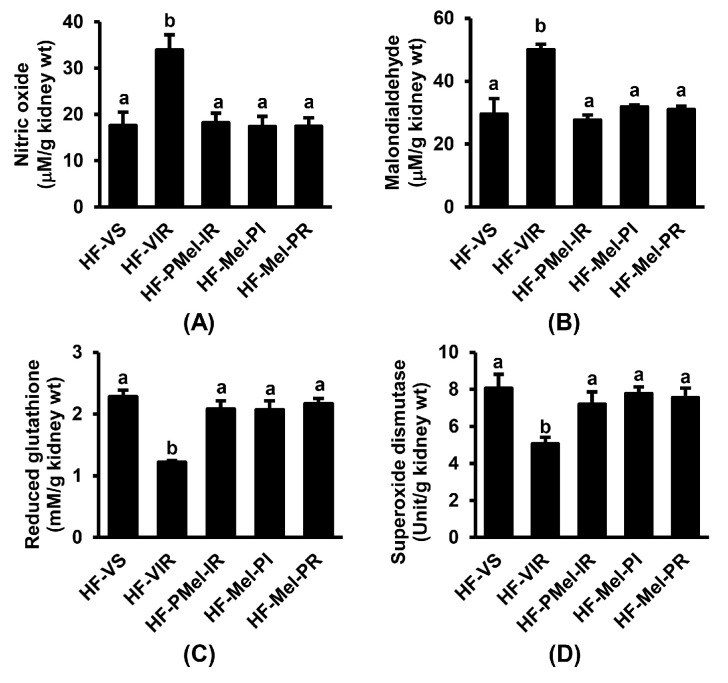
Effects of melatonin treatment on renal oxidative stress following ischemia–reperfusion (IR) in obese rats. (**A**) Nitric oxide; (**B**) malondialdehyde; (**C**) reduced glutathione; (**D**) superoxide dismutase. Values are means ± SEM (n = 6 each). HF-VS: high-fat-diet-fed rats and a vehicle-treated sham group; HF-VIR: high-fat-diet-fed rats and a vehicle-treated IR group; HF-PMel-IR: high-fat-diet-fed rats and melatonin pretreatment 4 weeks before IR induction; HF-Mel-PI: high-fat-diet-fed rats and melatonin treatment 5 min before ischemia; HF-Mel-PR: high-fat-diet-fed rats and melatonin treatment 5 min before reperfusion. Different lowercase letters denote statistical differences at *p* < 0.05 between groups.

To determine whether the amelioration of renal IR injury by melatonin in HFD-fed rats was related to oxidative modification, renal oxidative stress markers were evaluated. As shown in Figure 3, the kidney tissue levels of NO and MDA in the HF-VIR group were significantly increased, while the levels of GSH and SOD were significantly decreased in comparison with the HF-VS group. All these changes were significantly restored upon treatment with melatonin. The amelioration by melatonin was evident at all points of administration during the incidence of IR.

### 3.4. Melatonin Protects against Renal IR-Induced Mitochondrial Dysfunction and Ultrastructural Damage in Obese Rats

**Figure 4:**
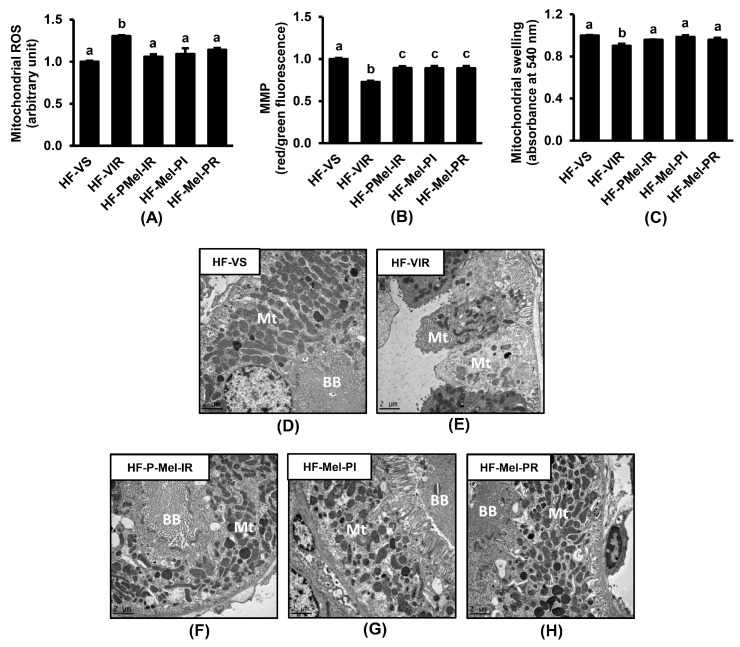
Effects of melatonin treatment on renal mitochondrial function and ultrastructural changes following ischemia–reperfusion (IR) in obese rats. (**A**) Mitochondrial reactive oxygen species production; (**B**) mitochondrial membrane potential changes; (**C**) mitochondrial swelling; and (**D**–**H**) transmission electron micrographs of proximal tubules (original magnification 3000×) showing loss of brush borders (BB), abnormal mitochondria (Mt), and reduced Mt numbers in the HF-VIR group. Values are means ± SEM (n = 6 each). HF-VS: high-fat-diet-fed rats and a vehicle-treated sham group; HF-VIR: high-fat-diet-fed rats and a vehicle-treated IR group; HF-PMel-IR: high-fat-diet-fed rats and melatonin pretreatment 4 weeks before IR induction; HF-Mel-PI: high-fat-diet-fed rats and melatonin treatment 5 min before ischemia; HF-Mel-PR: high-fat-diet-fed rats and melatonin treatment 5 min before and 6 h after reperfusion; ROS: reactive oxygen species; MMP: mitochondrial membrane potential. Different lowercase letters denote statistical differences at *p* < 0.05 between groups.

Renal IR resulted in mitochondrial dysfunction, as observed by significant increases in mitochondrial ROS production, the dissipation of the mitochondrial membrane potential, and mitochondrial swelling in the HF-VIR group compared to the HF-VS group (Figure 4A–C). Electron microscopic images provided further support to the mitochondrial functional abnormalities (Figure 4D–H). The loss of brush borders, cytoplasmic vacuolization, disruption of the basement membrane with tubular luminal debris, mitochondrial swelling, less dense and abnormal cristae, cellular necrosis, and reduced mitochondrial numbers were all evident in the HF-VIR group. The administration of melatonin to the obese rats at 4 weeks before IR induction, at 5 min before renal ischemia, or at the onset of reperfusion obviously reduced all ultrastructural damage caused by renal IR.

### 3.5. Melatonin Improves Signal Transduction Disruption of the AMPK-PGC-1α-SIRT3-SOD2 Axis Caused by Renal IR in Obese Rats

**Figure 5:**
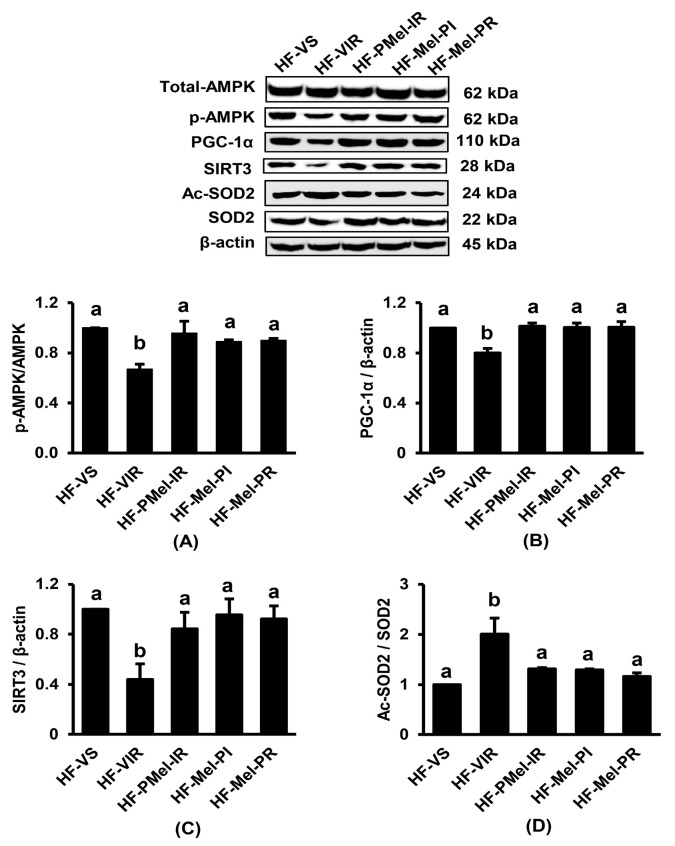
Effects of ischemia–reperfusion (IR) and melatonin treatment in obese rats on renal cortical expression of proteins involved in AMPK-PGC-1α-SIRT3-SOD2 axis. (**A**) p-AMPK/AMPK; (**B**) PGC-1α/β-actin; (**C**) SIRT3/β-actin; (**D**) Ac-SOD2/SOD2. Values are means ± SEM (n = 3 each). HF-VS: high-fat-diet-fed rats and a vehicle-treated sham group; HF-VIR: high-fat-diet-fed rats and a vehicle-treated IR group; HF-PMel-IR: high-fat-diet-fed rats and melatonin pretreatment 4 weeks before IR induction; HF-Mel-PI: high-fat-diet-fed rats and melatonin treatment 5 min before ischemia; HF-Mel-PR: high-fat-diet-fed rats and melatonin treatment 5 min before reperfusion. Different lowercase letters denote statistical differences at *p* < 0.05 between groups.

As shown in Figure 5A–C, Western blot analysis revealed that the protein expression of p-AMPK/AMPK, PGC-1α, and SIRT3 was significantly decreased in obese rats that underwent renal IR induction. A significant increase in the Ac-SOD2/SOD2 ratio, caused by the increased expression of Ac-SOD2 and decreased SOD2, was also observed in the HF-VIR group compared to the HF-VS group (Figure 5D). Treatment with melatonin in all HFD-fed-IR groups significantly improved this disturbance to these levels, which were comparable to those in the HF-VS controls.

### 3.6. Melatonin Attenuates Renal IR-Induced Mitochondrial Dynamics and Mitophagy Imbalance in Obese Rats

**Figure 6:**
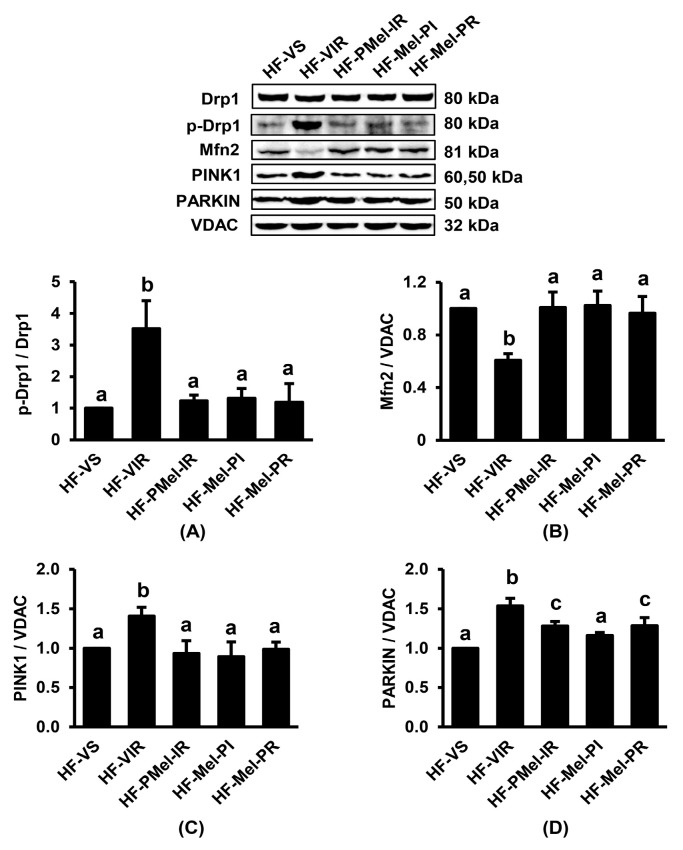
Effects of ischemia–reperfusion (IR) and melatonin treatment in obese rats on renal cortical expression of proteins involved in mitochondrial dynamics and mitophagy. (**A**) p-Drp1/Drp1; (**B**) Mfn2/β-actin; (**C**) PINK1/VDAC; (**D**) PARKIN/VDAC. Values are means ± SEM (n = 3 each). HF-VS: high-fat-diet-fed rats and a vehicle-treated sham group; HF-VIR: high-fat-diet-fed rats and a vehicle-treated IR group; HF-PMel-IR: high-fat-diet-fed rats and melatonin pretreatment 4 weeks before IR induction; HF-Mel-PI: high-fat-diet-fed rats and melatonin treatment 5 min before ischemia; HF-Mel-PR: high-fat-diet-fed rats and melatonin treatment 5 min before reperfusion. Different lowercase letters denote statistical differences at *p* < 0.05 between groups.

Renal IR resulted in a significant increase in p-Drp1/Drp1 and a decrease in Mfn2 in HF-VIR rats compared to HF-VS rats (Figure 6A,B). The HF-VIR rats also demonstrated significant increases in the expression of PINK1 and PARKIN after IR incidence (Figure 6C,D). Abnormalities in proteins associated with mitochondrial dynamics and mitophagy were significantly decreased in all HF-IR groups after melatonin treatment, regardless of the administration time.

### 3.7. Melatonin Reduces Renal IR-Induced Apoptosis in Obese Rats

**Figure 7:**
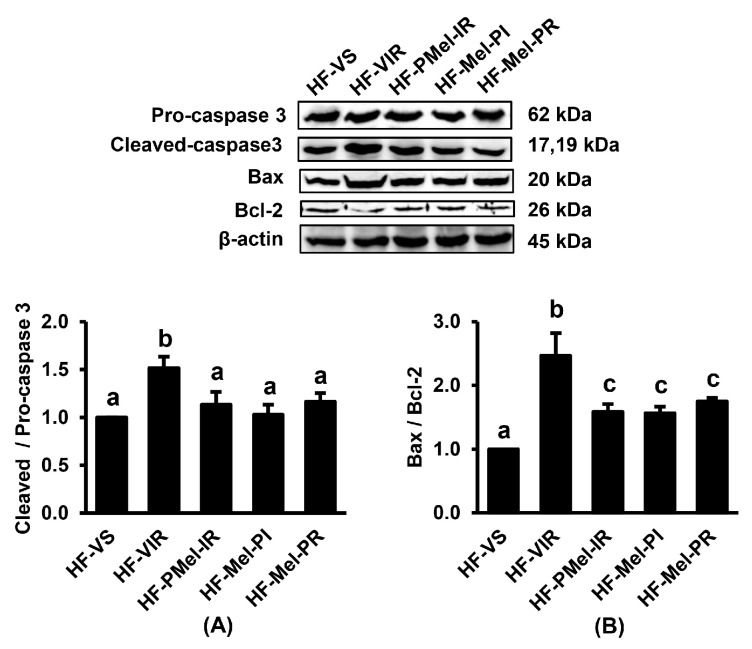
Effects of ischemia–reperfusion (IR) and melatonin treatment in obese rats on renal cortical expression of proteins involved in apoptosis. (**A**) Cleaved/pro-caspase 3; (**B**) Bax/Bcl-2. Values are means ± SEM (n = 3 each). HF-VS: high-fat-diet-fed rats and a vehicle-treated sham group; HF-VIR: high-fat-diet-fed rats and a vehicle-treated IR group; HF-PMel-IR: high-fat-diet-fed rats and melatonin pretreatment 4 weeks before IR induction; HF-Mel-PI: high-fat-diet-fed rats and melatonin treatment 5 min before ischemia; HF-Mel-PR: high-fat-diet-fed rats and melatonin treatment 5 min before reperfusion. Different lowercase letters denote statistical differences at *p* < 0.05 between groups.

The expression of apoptotic proteins cleaved/pro-caspase 3 and Bax/Bcl-2 was significantly upregulated in the HF-IR group compared with the HF-VS group (Figure 7). However, all these changes were significantly attenuated upon treatment with melatonin at different time points during the IR period.

## 4. Discussion

The major issue considered in this study was the consequences of acute kidney injury caused by renal ischemia and reperfusion in obesity and, most importantly, the possible beneficial effects of melatonin in this condition. Both the preventive and therapeutic potential of melatonin were tested by the administration of melatonin at various time points during an ischemia–reperfusion episode. Our study demonstrated the exacerbation of kidney injury after ischemia–reperfusion in the obese rats and provided evidence to indicate that melatonin is effective as both a preventive and therapeutic agent to improve acute kidney injury caused by ischemia and reperfusion in obese conditions through the maintenance of homeostasis and integrity within the mitochondria.

Obesity is defined as abnormal or excessive fat accumulation that presents a risk to health. It is now dramatically rising and has become a serious public health problem of the 21st century. The consumption of a HFD is common in many countries and studies have reported that HFD consumption can induce renal lipotoxicity and metabolic disturbances that can compromise the vital functions of the kidney [24]. Therefore, we chose HFD-induced obesity as a representative model for the investigation. After 16 weeks of HFD intake, all HFD-fed rats successfully developed obesity as manifested by the presence of overweight and hyperlipidemia in comparison to the age-matched rats that received a normal diet.

Epidemiological studies support the increased prevalence of acute renal disease in patients consuming a HFD [8]. Recent studies have also shown that long-term HFD consumption increases the susceptibility of renal tissue to ischemic injury [24]. Our results are consistent with these reports, as seen by the degree of renal functional disorder (increased blood urea nitrogen and creatinine, reduced creatinine clearance) and histopathological damage in HFD-fed rats after acute IR incidence, which were more intense than those detected in ND-fed rats. Interestingly, we found that IR-associated renal dysfunction in HFD-fed rats was significantly ameliorated upon melatonin treatment. This improvement was evident irrespective of the time of melatonin administration, indicating that melatonin possessed both prophylactic and therapeutic benefits against renal IR injury in the obese condition.

Oxidative stress is recognized as an important component involved in the pathophysiological processes observed during ischemia and, particularly, reperfusion injury [3]. We determined whether the benefits of melatonin were linked to its abilities to cope with oxidative stress generation. As expected, our results showed that the increases in the kidney tissue levels of NO and MDA, along with decreases in the antioxidant levels of GSH and SOD, in obese rats after IR injury were all restored upon treatment with melatonin. These findings simply indicate that the effectiveness of melatonin in regulating oxidative balance is mediated by its well-established properties as a potent free radical scavenger as well as antioxidant.

Mitochondria are also a major source of ROS production, aside from their crucial role in ATP production. Mitochondrial oxidative stress and dysfunction is accepted as an important underlying mechanism responsible for oxidant-induced disorder in several organ systems, including ischemia–reperfusion of the kidney [3]. To maintain homeostasis and integrity within the mitochondria, several important processes, e.g., mitochondrial redox balance, bioenergetics, biogenesis, dynamics, and mitophagy, need to be tightly controlled [3].

In this study, IR-induced renal injury in obese rats was associated with the existence of mitochondrial dysfunction, as demonstrated by the increased mitochondrial ROS production, the disruption of the mitochondrial membrane potential, and the swelling of mitochondria. Excessive ROS directly damages the mitochondrial respiratory chain, especially complexes I and III, leading to increased electron leakage and more free radical production [25]. The overproduction of ROS also damages the structure of the mitochondrial membrane and induces the opening of mitochondrial permeability transition pores (mPTP), resulting in the loss of membrane potential [26]. Increasing mitochondrial permeability leads to mitochondrial swelling and rupture, which increases the release of pro-apoptotic factors into the cytoplasm and apoptosis induction, as shown by the increased expression of cleaved/pro-caspase 3 as well as Bax/Bcl-2 in the obese-IR group. Our electron micrographs further demonstrated mitochondrial fragmentation and a mitochondrial number reduction in the obese-IR group, which corresponded to the results from the Western blot showing the increased expression of p-Drp1/Drp1, decreased Mfn2, and increased PINK1 and PARKIN. All these findings signified a disturbance in mitochondrial redox balance, bioenergetics, biogenesis, dynamics, and mitophagy initiated by renal IR in the HFD-induced obese rats.

The AMPK-PGC-1α-SIRT3-SOD2 axis has been recognized as essential for the regulation of mitochondrial homeostasis and integrity. SIRT3, the primary mitochondrial NAD^+^-dependent protein deacetylase, plays a key role in this process. It is acknowledged that SIRT3 is a crucial metabolic sensor that regulates ATP generation and mitochondria’s adaptive response to stress [27]. SIRT3 powerfully boosts ATP levels by directly binding to and regulating complexes I, II, and V of the electron transport chain. It manages the molecular machinery that governs mitochondrial dynamic and permeability [27]. SIRT3 also plays a major role in mitochondrial redox control via the deacetylation of SOD2 and thus the activation of mitochondrial antioxidant enzyme SOD2 [28]. In addition, SIRT3 activates isocitrate dehydrogenase 2 (IDH2) to promote the restoration of antioxidants, especially glutathione, and catalyzes a key step of the tricarboxylic acid cycle [27]. SIRT3 has also been described as controlling the microtubule network-dependent trafficking of functional mitochondria between renal tubular epithelial cells, a process that preserves the proper cellular bioenergetic profile and antioxidant defense [27]. AMPK is a crucial cellular energy sensor that plays a major role in the regulation of signaling pathways that replenish cellular ATP supplies. AMPK acts as a signal integration platform to maintain mitochondrial health by simultaneously regulating mitochondrial fission, mitophagy, and the transcription control of mitochondrial biogenesis [7,29]. PGC-1α, a master regulator of mitochondrial biogenesis and function, also plays a role in the regulation of oxidative phosphorylation, fatty acid/lipid metabolism, and the modulation of ROS [30]. Importantly, a recent study provided evidence to indicate that SIRT3 can activate AMPK-related mitochondrial biogenesis, while AMPK can control the transcription and activity of PGC-1α [31].

Interestingly, our study showed that melatonin treatment in obese-IR rats considerably attenuated mitochondrial dysfunction and all the disturbances in mitochondrial redox balance, bioenergetics, biogenesis, dynamics, and mitophagy. Our study also demonstrated that the benefits of melatonin were associated with the correction of the abnormal expression of proteins involved in the AMPK-PGC-1α-SIRT3-SOD2 axis. Melatonin is documented as a mitochondrial protective agent, being able to act through both membrane-receptor-dependent and receptor-independent pathways to modulate several signaling molecules involved in mitochondrial quality and quantity control [32]. The contribution of melatonin in maintaining mitochondrial redox homeostasis by influencing the tricarboxylic acid cycle, mitochondrial oxidative phosphorylation, SIRT3, and SOD2 has recently been revealed [32]. Consistent with our findings, a recent study has shown the therapeutic potential of single-dose melatonin in the attenuation of cardiac IR injury in prediabetic obese rats through its ability to improve mitochondrial function and rebalance the mitochondrial dynamics [20].

Based on the data obtained from our study, including all available information presented above, it is suggested that melatonin exerts both prophylactic and therapeutic benefits to improve renal injury following ischemia and reperfusion in HFD-induced obese rats, possibly via activation of the AMPK-PGC-1α-SIRT3-SOD2 axis, and thus the maintenance of mitochondrial homeostasis and integrity. However, our work had certain limitations, as we were unable to precisely determine whether melatonin exerted its renoprotection via the melatonin receptor or a non-receptor pathway. More research utilizing either a nonselective or a specific (M1 or M2) melatonin receptor blocker will not only help in the discovery of new drugs for further development and translation to clinical usage, but also in the exploration of the pathogenic pathways that are active during renal IR injury in obesity.

## 5. Conclusions

The present study revealed the aggravation of renal injury after ischemia and reperfusion in obesity and demonstrated that melatonin, regardless of the administration time of treatment, is effective as a prophylactic and therapeutic intervention for renal ischemia and reperfusion injury in obese individuals. The benefits of melatonin appear to be mediated by its ability to modulate various signals within mitochondria that maintain mitochondrial redox balance and lead to the preservation of mitochondrial homeostasis and integrity. The study outcomes may be applied as a strategy to improve the prognosis of AKI induced by renal ischemia and reperfusion in the obese population.

## Figures and Tables

**Table 1:** Metabolic parameters in high-fat-diet-induced obese rats.

Parameters	Week 0		Week 16	
	ND	HFD	ND	HFD
Body weight (g)	190.67 ± 2.98	189.60 ± 1.33	445.42 ± 8.54 ^†^	565.00 ± 5.72 *^†^
Serum triglycerides (mg/dL)	62.12 ± 3.19	60.40 ± 2.28	88.26 ± 1.17 ^†^	102.81 ± 1.08 *^†^
Serum total cholesterol (mg/dL)	76.06 ± 6.00	75.56 ± 4.14	107.52 ± 1.47 ^†^	142.02 ± 3.98 *^†^

Values are mean ± SEM (ND: n = 12; HFD: n = 30). ND: normal-diet-fed rats; HFD: high-fat-diet-fed rats. * *p* < 0.05 vs. ND within the same week, ^†^ *p* < 0.05 vs. their respective values in week 0.

## Data Availability

The datasets used and/or analyzed during the current study are available from the corresponding author on reasonable request.
